# Text-Enhanced Graph Attention Hashing for Cross-Modal Retrieval

**DOI:** 10.3390/e26110911

**Published:** 2024-10-27

**Authors:** Qiang Zou, Shuli Cheng, Anyu Du, Jiayi Chen

**Affiliations:** College of Computer Science and Technology, Xinjiang University, Urumqi 830046, China; zouq@stu.xju.edu.cn (Q.Z.); anydxju@xju.edu.cn (A.D.); 107552203982@stu.xju.edu.cn (J.C.)

**Keywords:** cross-modal hashing, graph attention, feature fusion, vision transformer, information entropy

## Abstract

Deep hashing technology, known for its low-cost storage and rapid retrieval, has become a focal point in cross-modal retrieval research as multimodal data continue to grow. However, existing supervised methods often overlook noisy labels and multiscale features in different modal datasets, leading to higher information entropy in the generated hash codes and features, which reduces retrieval performance. The variation in text annotation information across datasets further increases the information entropy during text feature extraction, resulting in suboptimal outcomes. Consequently, reducing the information entropy in text feature extraction, supplementing text feature information, and enhancing the retrieval efficiency of large-scale media data are critical challenges in cross-modal retrieval research. To tackle these, this paper introduces the Text-Enhanced Graph Attention Hashing for Cross-Modal Retrieval (TEGAH) framework. TEGAH incorporates a deep text feature extraction network and a multiscale label region fusion network to minimize information entropy and optimize feature extraction. Additionally, a Graph-Attention-based modal feature fusion network is designed to efficiently integrate multimodal information, enhance the affinity of the network for different modes, and retain more semantic information. Extensive experiments on three multilabel datasets demonstrate that the TEGAH framework significantly outperforms state-of-the-art cross-modal hashing methods.

## 1. Introduction

In the era of information explosion, data take on various forms, including text, images, and videos, across different modalities. These diverse modalities of data have accumulated massive information resources in areas such as the internet, multimedia retrieval, and social networks. However, effectively organizing, retrieving, and understanding data from different modalities pose a significant challenge in the field of information retrieval [1]. Cross-modal retrieval aims to address this issue by establishing semantic links between data of different modalities, enabling users to search for one modality of data (such as text) with another (such as images) [2]. To achieve efficient cross-modal retrieval, hashing methods are widely applied in the indexing and retrieval processes of cross-modal data [3,4]. Cross-modal hash retrieval learns a common hash function to map data from different modalities into the same hash space, ensuring that even data from different modalities can be mapped to similar hash codes as long as they share semantic content. This method effectively bridges the semantic gap between different modalities, realizing fast and accurate cross-modal data retrieval [5,6,7,8].

The development of Convolutional Neural Networks (CNN) in recent years has significantly enhanced the performance of cross-modal hash retrieval. CNNs, with their powerful nonlinearity and translation invariance, enable the extraction of higher-quality features from different modalities [9,10]. However, the semantic gap between different modalities is an inherent issue. Images contain more semantic features than text and usually offer richer high-level features. Despite this, most CNN-based cross-modal hashing works [11,12,13,14,15] employ the same loss function for feature learning across modalities. Such approaches, however, overlook the differences in information between modalities and do not adequately address the issue of semantic discrepancies [16,17,18,19,20,21,22]. Therefore, reducing this semantic gap to enhance the representation of weaker modal features becomes particularly crucial.

Current research is delving deeper into methods for cross-modal hash learning, yet traditional approaches in this field still face numerous challenges in both theory and application. Methods cited in references [23,24,25] aim to exploit the high-order semantic associations among data’s multilabels and utilizing label information to extract implicit semantic content. However, these methods overlook the prior knowledge contained in label information, specifically the weight information of labels. This oversight results in an inability to effectively enhance retrieval performance. Methods referenced in [26,27,28,29] employ the fusion of multiscale features from different modalities for semantic optimization, make the more compact between the hash codes. Additionally, they align similar semantic features across modalities using loss functions that incorporate semantic optimization, which improves retrieval performance. However, these approaches do not optimize the cross-modal retrieval process from a textual standpoint, offering limited consideration for text features and thereby failing to effectively integrate features from different modalities. Approaches cited in [30,31,32,33] utilize unsupervised clustering to optimize the extraction of intrinsic similarity structures between modalities, enhancing their integration through semantic alignment. However, these methods ignore the structural discrepancies between textual and visual semantics, struggling to effectively align and merge semantic features from different modalities.

With the widespread application of Transformers [34] in the visual domain, an increasing number of cross-modal hash retrieval methods have started to incorporate Transformer models, such as Swin-Transformer [35]. The continuous development of these Transformer-based variants has made Transformer models more suitable for image tasks. Consequently, leveraging the powerful performance of Transformers for retrieval tasks has significantly enhanced cross-modal retrieval performance  [36,37,38,39]. However, these cross-modal hash retrieval methods, based on both convolutional neural networks and Transformers, have not addressed the issue of textual semantic information scarcity and the severe imbalance between textual data and visual data. Merely optimizing from the image perspective struggles to rectify the inherent issues within text.

**Figure 1:**
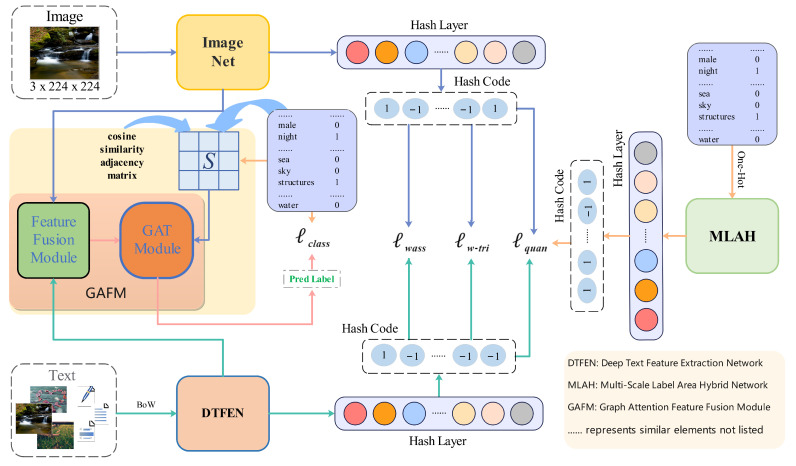
The overall framework of TEGAH can be divided into five parts: (1) Image-Net: employing the Swin Transformer-Small (SwinT-S) model to extract semantic features from images and map these features into the feature space; (2) Graph Attention Feature Fusion Module (GAFM): a feature fusion and alignment network that weights and merges image and text features to address semantic discrepancies between different modalities; (3) Multiscale Label Area Hybrid Network (MLAH): utilizing multiscale features across four layers and incorporating multiscale attention to mitigate issues related to insufficient textual information; (4) Deep Text Feature Extraction Network (DTFEN): improving upon traditional methods by capturing high-quality textual feature information; (5) Hash Learning Module: transforming features into hash codes through nonlinear changes, with training assisted by a combination of cosine-weighted triplet loss, label distillation loss, Wasserstein loss, and quantization loss, each component specifically designed to enhance the extraction, fusion, and representation of multimodal features, thereby improving the accuracy and efficiency of cross-modal hash retrieval.

Addressing the aforementioned issues, this paper introduces a Text-Enhanced Graph Attention Hashing for Cross-Modal Retrieval (TEGAH) framework. This framework employs Graph Attention (GAT) [40] combined with a multiscale approach for modal feature fusion, effectively mitigating semantic loss and discrepancies caused by the fusion of different modal information. Additionally, it leverages label information to supplement and enhance textual information. As far as we know, GAT is not used in cross-modal hash retrieval to realize the relevant application of feature fusion, but GAT or GCN is more used for feature extraction or classification [41,42]. This paper is the first to introduce the GAT into cross-modal hash retrieval for cross-modal feature fusion, effectively generating pseudo-labels that incorporate features from different modalities, thereby achieving efficient cross-modal hash retrieval. As illustrated in Figure 1, the overall architecture of this paper comprises four parts: an image network, a text network, a label network, and a feature fusion network, collectively referred to as the cross-modal feature fusion learning network. The image network utilizes a Transformer architecture image encoder to model long-distance visual dependencies of images and capture their global information. In the text network, two deep feature extraction modules and an autoencoder are designed for text feature learning, with each text being transformed into a Bag-of-Words (BoW) vector. To alleviate the scarcity of textual information, a novel Multiscale Label Area Hybrid Network (MLAH) is proposed, focusing on supplementing text with multilabel information. This network builds attention graphs for sparse labels and performs multiscale feature extraction to capture their global dependencies, enriching text feature information and optimizing the semantic feature extraction effect of the text network. Finally, the graph attention feature fusion module (GAFM) proposed in this paper deeply merges and aligns the acquired image semantic features and text semantic features, which can dynamically adjust the similar structure between different modal nodes and generate better prediction labels to supplement the whole network. This framework effectively reduces the semantic discrepancies between different modalities, to some extent enhancing the feature representation capability of different modalities for better semantic alignment and fusion, resulting in higher quality binary hash codes.

The main contributions of our work are as follows:

- The Text-Enhanced Graph Attention Hashing for Cross-Modal Retrieval (TEGAH) framework proposed in this paper marks the first instance of integrating graph attention to achieve cross-modal feature fusion, and a Graph Attention Feature Fusion Module (GAFM) was designed for deep fusion between different modal features to solve the semantic divergence problem in cross modal hash retrieval. It facilitates better semantic alignment and feature fusion between different modalities and, to some extent, compensates for semantic losses incurred during the fusion process, thereby enhancing the performance of the network.
- The paper introduces a Multiscale Label Area Hybrid Network (MLAH), which mitigates the issue of sparse label distribution by drawing closer the sparsely distributed label information and fully exploring their interconnections. This approach reduces the model’s misinterpretation of the semantic relevance of different labels caused by sparse label distribution. Additionally, by fully extracting multilabel features at different scales, MLAH adds multigranularity feature representations to textual features, thereby enhancing the expressive capability of textual features.
- The paper proposes a Deep Text Feature Extraction Network (DTFEN) that modifies the text network by incorporating deep feature extraction modules and an autoencoder, as opposed to the conventional use of fully connected layers for extracting text information. By employing a deep feature extraction module prior to the hash function, it more effectively integrates deep textual features, thereby improving the utilization rate of text features.

The remainder of this paper is organized as follows. Section 2 provides an overview of work related to cross-modal hashing methods. Section 3 details our Text-Enhanced Graph Attention Transformer for Hash-based Cross-Modal Retrieval (TEGAH) method. Section 4 presents our experimental results and analysis. Finally, Section 6 offers our conclusions.

## 2. Related Work

Hashing methods have garnered widespread attention in retrieval scenarios. Cross-modal hashing methods learn hash functions to map high-dimensional information from diverse modalities into a unified common space, minimizing the semantic gaps between modalities. This ensures that the Hamming distance between semantically related data is smaller than that between semantically unrelated data. In this section, we briefly review supervised and unsupervised cross-modal hashing methods, as well as the work involving Transformer and GAT in cross-modal hash retrieval.

### 2.1. Supervised Cross-Modal Hashing

Supervised cross-modal hash methods enable efficient and accurate retrieval by utilizing label information or inter-modal pairing information to map data from different modalities into a shared hash space. The essence of these methods lies in effectively leveraging available supervisory information to guide the hash encoding process, ensuring the semantic consistency across modalities is maintained. DCMH [5] is a prime example, utilizing AlexNet [9] as the foundation for feature extraction and combining it with fully connected layers for learning features and hash codes, facilitating effective retrieval between images and texts. DVSH [1] seeks to enhance semantic matching across modalities by integrating the textual semantics of images. PRDH [28] introduces inter-modal and decorrelation losses to optimize the similar structure across modalities. CMHH [6] employs a Bayesian method for joint optimization, finely tuning the losses in the quantization process. HSCH [29] explores fine-grained data information to avoid semantic conflicts and preserve important similarity features. DJSAH [26] ensures high-level discriminative semantics are preserved in the hash codes through semantic alignment and latent representations in a shared latent space. SSCH [30] learns hash representations for various data through an alignment-free pseudo-label process and label enhancement strategy. MAFH [24] adopts a collective matrix decomposition method to map kernelized features of different modalities to a shared latent space, optimizing hash code length through semantic labels for bit scalability. DAPH [18], GCDH [42], and MIAN [19] propose their optimization strategies, such as novel hash loss, GCN, and a probabilistic modality alignment framework, refining features and optimizing cross-modal hash retrieval performance from different perspectives. SCCGDH [20] and MESDCH [25] further enhance the robustness and discriminability of hash encoding through category center hash functions and multilabel modality-enhanced attention modules.

### 2.2. Unsupervised Cross-Modal Hashing

Unsupervised cross-modal hashing methods do not utilize real labels but instead learn hash functions by discovering the inherent similarities within the data. For example, UCMH [39] enhances retrieval performance by optimizing a novel hash-similarity friendly loss. It initially trains a Modality Interaction Enabled (MIE) similarity generator to produce a superior MIE similarity matrix for the training set. Then, it uses the generated MIE similarity matrix to guide the training of a deep hash network, introducing a novel bit selection module that interacts between continuous codes of different modalities to generate high-quality unified binary codes for the quantization loss, thereby further improving retrieval performance. DAEH [32] designs an Adaptive Teacher-Guided Enhancement (ATGE) optimization strategy, utilizing information theory to identify weaker hash functions. UKD [33] introduces a new cross-modal hash distillation method, allowing supervised methods to be guided by the outputs produced by unsupervised methods. UCCH [22] incorporates contrastive learning into cross-modal hash retrieval, introducing a novel momentum optimizer that enables the binary hash function to learn, thus bridging the gap between contrastive learning and hashing algorithms. To overcome the False Negative Pair (FNP) challenge, UCCH proposes a Cross-Modal Ranking Learning Loss (CRL), leveraging all pairs instead of hard negative pairs for better performance and robustness.

### 2.3. Transformer-Based Cross-Modal Hashing

With the widespread application of Transformers in both vision and text, Transformer-based cross-modal hashing employs Transformers to learn the intrinsic similarities between images and texts for hash function learning. For example, DCHMT [36] introduces a selection mechanism to generate hash codes, transforming the discrete space into a continuous one. Hash codes are encoded as a series of 2D vectors. UCMFH [37] is the first to explore the effectiveness of the CLIP [43] model in cross-modal hash retrieval, proposing a simple yet powerful baseline model. It utilizes the CLIP model to extract textual and visual features, then generates hash codes through contrastive learning and multimodal fusion. However, it employs a simple weighted averaging method, not fully considering the semantic alignment and complementarity between text and images. DSPH [23] proposes a novel semantic-aware proxy loss for training a MIE similarity generator, creating a superior MIE similarity matrix for the training set. It then uses this matrix as guidance to train a deep hash network, with two Transformer encoders serving as feature extractors for images and texts.

### 2.4. Graphical Attention Network

The Graph Attention (GAT) Network is a graph neural network based on the self-attention mechanism. MS2GAH [41] builds graph features using the adjacency of nodes and allocates varying weights to neighboring edges to bolster the model’s resilience. It further employs multilabel annotations to connect the semantic relevance across modalities with greater detail. Introducing the GAT network into cross-modal hash retrieval enables the effective learning of representations for graph-structured data. GAT leverages the structural information of heterogeneous graphs to build image and text data in a unified space, thus capturing the high-level semantic relationships between data more effectively. Through multilayer graph attention networks aggregating neighbor features, the expressive power of each node is enhanced, and different weights can be adaptively allocated to different neighbors. GAT can train the model with adversarial loss and triplet loss, achieving personalized cross-modal retrieval, and enhancing the accuracy and efficiency of retrieval.

## 3. Methodology

In this section, we will look at TEGAH in detail. See Section 3.1 for definitions. The details of the Graph Attention Feature Fusion Module (GAFM) are described in Section 3.2. Multiscale Label Area Hybrid networks (MLFW) and deep text feature extraction networks are described in more detail in Section 3.3 and Section 3.4. Section 3.5 shows the details of the TEGAH model loss function, and in Section 3.6, we detail the various state-of-the-art (SOTA) methods we compared and the parameter settings of the datasets.

### 3.1. Formula Definition

Similar to most other approaches, in this paper, the cross-modal hash retrieval framework utilizes image-text-label triples as input, assuming there are *N* pairs of image-text data and their labels. Typically, these triples can be represented as ${\left\{\left(X_{n},Y_{n},Z_{n}\right)\right\}}_{n=1}^{N}$, where *X* denotes images, *Y* denotes texts, and *Z* represents labels. This paper uses sets to represent these three types of information, i.e., the image set $X=\left\{x_{1},x_{2},\ldots ,x_{N}\right\}$, the text set $Y=\left\{y_{1},y_{2},\ldots ,y_{N}\right\}$, and the label set $Z=\left\{z_{1},z_{2},\ldots ,z_{N}\right\}$, where $Z_{n}\in {\left\{0,1\right\}}^{C}$ represents a One-Hot encoding, *C* stands for the number of label categories, and $z_{n}=1$ if the image and text samples belong to this class; otherwise, $z_{n}=0$. The aforementioned information can be defined as a set of triples $S=\left\{\left(x_{j},y_{j},z_{j}\right)|x_{j}\in X,y_{j}\in Y,z_{j}\in Z\right\}$, which contains all triples of images, texts, and labels. The features extracted through the image network, text network, label network, and cross-modal feature fusion network are denoted as $F_{i}^{k}\in R^{k},i\in \left\{x,y,z\right\}$, mapping different modal features to the same *k*-dimensional feature space through the mentioned networks. The overall architecture of our proposed TEGAH framework is shown in Figure 1. The hash codes are represented as $b_{x}\in {\left\{-1,1\right\}}^{M}$, $b_{y}\in {\left\{-1,1\right\}}^{M}$, and $b_{z}\in {\left\{-1,1\right\}}^{M}$, where *M* denotes the length of the hash code, using H(·) to denote the hash function, and Tanh function to map different modal features to the corresponding hash code length. The Tanh function can be represented as follows:

(1) $$\begin{array}{c} Tanh\left(t\right)=\frac{e^{x}-e^{-x}}{e^{x}+e^{-x}} \end{array}$$

The sign function is used to generate the corresponding binary hash codes, typically denoted as sign. Its definition is as follows:

(2) $$\begin{array}{c} sign\left(t\right)=\left\{\begin{array}{cc} 1, & if\;t\geq 0\\ -1, & if\;t<0 \end{array}\right. \end{array}$$

### 3.2. Graph Attention Feature Fusion Module (GAFM)

The Graph Attention Network (GAT) is a graph neural network based on the self-attention mechanism, enabling the utilization of structural information from heterogeneous graphs. By constructing image and text data within a unified space, GAT can effectively capture high-level semantic relationships between data. Through the aggregation of neighbor features by multilayer graph attention networks, the expressive capability of each node is enhanced, allowing for the adaptive allocation of different weights to different neighbors. Moreover, GAT leverages the self-attention mechanism to adaptively weight different modal data, generating more accurate pseudo-labels. This assists in addressing the issue of insufficient cross-modal data labeling, thereby improving the model’s generalization ability. In summary, GAT is an effective method for integrating image and textual features, enhancing the performance of cross-modal hash retrieval.

Some existing methods utilize GCN as a feature extractor to extract features from different modalities, which can degrade retrieval performance. In contrast, by weighting the features from different modalities through fusion, the generated pseudo-labels can, to some extent, compensate for the lack of richness in textual data, serving as a supplement to textual information.

**Figure 2:**
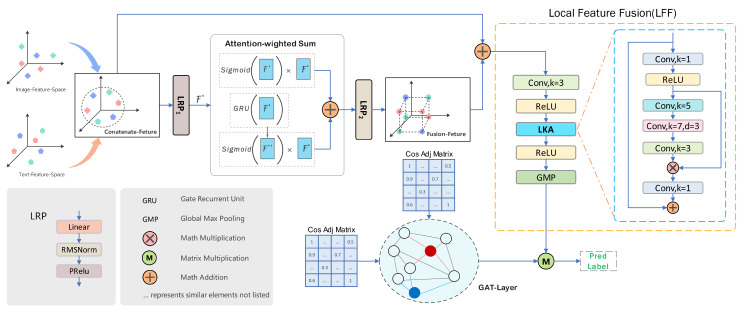
Graph Attention Feature Fusion Module (GAFM) architecture integrates and aligns image and text features through the interaction of Layerwise Propagation Rule (LPR) and Gated Recurrent Unit (GRU), employing Local Linear Fusion (LLF) to mine multiscale information internally. The features are ultimately fed into the GAT to generate predicted pseudo-labels.

In the method proposed in this paper, we have designed a Graph Attention Feature Fusion Module (GAFM). Unlike the original GAT network, which requires the additional generation of co-occurrence matrix information, our approach repurposes the adjacency matrix as the co-occurrence matrix through cosine quantization and weighting operations. To a certain extent, we can consider the label information as a type of weight matrix. By reusing the label matrix and adjacency matrix, feature fusion can optimize the retrieval process beyond just the training phase, enhancing retrieval effectiveness and efficiency. The structure of our Graph Attention Feature Fusion Module is detailed in Figure 2. The feature representation extracted by the Image Encoder (ImgEncoder) from the image network is denoted as ImgEncode, and the hash code obtained from the image network is represented as $H_{x}^{k}$, where *M* denotes the *M*-dimensional hamming space, as shown in Equation (1). Finally, we use H(·) to map the multiscale label fusion features obtained above to the hash code length we need, taking image features as an example:

(3) $$\begin{array}{c} H_{x}^{M}=H\left(F_{x}^{k}\right) \end{array}$$

where $H_{x}^{M}$ represents the image hash code, M represents the length of the hash code, and H(·) represents the hash function. Assume that the features extracted by the image feature extractor and text feature extractor are $F_{x}^{k}$ and $F_{y}^{k}$, respectively, and the features extracted by the label network are $F_{z}^{k}$, the adjacency matrix is defined as $A\in R^{C\times C}$, where C represents the category of the label number. We use two layers of GAT, and use Concat to combine different modal features. We perform deep feature fusion and optimization through the network as a whole, that is, $F_{c}=\mathrm{Concat}\left({F_{x}}^{k},F_{y}^{k}\right)$, and $F_{fusion}$ denotes the fused features and the overall formula of the modal feature fusion method, which is expressed as follows:

(4) $$\begin{array}{c} F_{fusion}=LFF\left(F_{c}+LRP_{2}\left(Attention_{g}\right)\right) \end{array}$$

Among them, the $LRP_{i}$ component is defined as follows:

(5) $$\begin{array}{c} LRP_{i}=PReLU\left(RMSNorm\left(Linear\left(\cdot \right)\right)\right)\\ s.t.\;i\in \{1,2,3,4\} \end{array}$$

We employ the PReLU activation function, Root Mean Square Layer Normalization (RMSNorm), and a Linear mapping function to maintain normalization and dimensional consistency of the fused features, ensuring the stability and generalization capability of the network.

To better fuse coarse-grained features and enhance the representational effect of the fused features, we propose a Local Feature Fusion Module (LFF) composed of Local Kernel Alignment (LKA), convolution, the activation function (ReLU), and Global Max Pooling (GMP). LKA provides multigranularity local fusion features for predicting labels, as illustrated in Figure 2. Additionally, we derive different weighted scores λ and *h* from features ${F_{c}}^{\prime \prime }$ and ${F_{c}}^{\prime }$ of varying depths for the purpose of weighted fusion, specifically expressed as follows:

(6) $$\begin{array}{c} \begin{array}{c} Attention_{g}=\lambda *{F_{c}}^{\prime \prime }+\hbar *{F_{c}}^{\prime }\\ s.t.\;\lambda =\sigma \left({F_{c}}^{\prime \prime }\right),\hbar =\sigma \left({F_{c}}^{\prime }\right) \end{array} \end{array}$$

to better integrate feature representations from different modalities, we employ the Gate Recurrent Unit (GRU) [44] for a deeper level of cross-modal fusion. The specific operations are as follows:

(7) $$\begin{array}{c} {F_{c}}^{\prime }=LRP_{1}\left(F_{c}\right)\\ {F_{c}}^{\prime \prime }=GRU\left(LRP_{1}\left(F_{c}\right)\right) \end{array}$$

the asterisk (*) represents the multiplication operation and σ(t) denotes the sigmoid function, which is expressed as:

(8) $$\begin{array}{c} \sigma \left(t\right)=\frac{1}{1+e^{-t}} \end{array}$$

where ${F_{c}}^{\prime }$ represents the feature vector obtained after the first feature depth fusion extraction layer $LRP_{1}$, and ${F_{c}}^{\prime \prime }$ denotes the features extracted after passing through both $LRP_{1}$ and the GRU. The GRU, widely utilized in Natural Language Processing (NLP), employs a gating mechanism to fuse information from different modalities, while also filtering out dissimilar features and retaining those with semantic relevance. It is evident that, throughout our feature fusion process, we achieve multilevel and multigranularity deep feature fusion and alignment, taking into account the global and local relevance within the fused features.

Given the GAT’s capacity for thorough exploration of label information, we utilize it as a classifier to merge with the fused features obtained from the above process to generate pseudo-labels. The weighted sum $h_{i}^{\prime }$ of node *i* and its adjacent node features *j* is determined by the normalized attention weight coefficients $e_{ij}$:

(9) $$\begin{array}{c} \begin{array}{c} h_{i}^{\prime }=\sum_{j=1}^{N}\frac{e_{ij}}{\sum_{k=1}^{N}e_{ik}}\left(Wh_{j}\right)\\ e_{ij}=\mathrm{LeakyReLU}\left(a^{T}\left[Wh_{i}∥Wh_{j}\right]\right) \end{array} \end{array}$$

where $h_{i}$ and $h_{j}$ represents the feature vector for node *i* and *j*, *W* denotes the weight matrix, *a* signifies the attention weight vector, which is a parameter that needs to be learned, LeakyReLU refers to a nonlinear activation function, and the concatenation operation of vectors is indicated by || and *N* represents the number of nodes. In GAT, each node *i* has a corresponding attentional weight $e_{ij}$ with every other node *j* to adjust the propagation of information. These attention weights are obtained through the linear combination of *a* and the node features, followed by normalization to ensure their sum equals 1.

As depicted in Figure 2, the weighted sum and weight parameters of $G^{l+1}$ are defined as $\Theta_{g}$. The hierarchical propagation rule of GAT can be defined as follows:

(10) $$\begin{array}{c} G^{l+1}=\varphi \left(e_{ij}A_{c}G^{l}W^{l}\right)\\ \tilde{\Omega }=F_{fusion}*{\left(F_{G}\right)}^{T} \end{array}$$

where *G* represents the pseudo-labels obtained through the GAT module and fusion module, φ represents the nonlinear operation, $\tilde{\Omega }$ signifies the predicted labels, $A_{c}$ stands for the adjacency matrix optimized through cosine similarity, $Z^{T}$ represents the transpose of label *Z*, and $F_{G}$ represents the weight set obtained from the adjacency matrix weighting calculation, which is calculated as follows:

(11) $$\begin{array}{c} \begin{array}{c} F_{G}=G^{l+1}\left(A_{c}|\Theta_{g}\right)\;\\ A_{c}=cos\left(Z^{T}\cdot Z,Z\cdot Z^{T}\right)=\frac{\left(Z^{T}\cdot Z\cdot Z^{T}\cdot Z^{T}\right)}{∥Z^{T}\cdot Z∥\times ∥Z^{T}\cdot Z∥} \end{array} \end{array}$$

We calculate the similarity probability score with the final value obtained through the GAFM and the real label. By comparing the similarity, we can balance the differences of different modal features, generate a better hash feature representation, and improve the retrieval effect. Label classification losses are calculated as follows:

(12) $$\begin{array}{c} L_{cls}=\sum_{i=1}\sum_{i=1}^{N}Z_{i}\log\left(\sigma \left(\tilde{\Omega }\right)\right)+\left(1-Z_{i}\right)\log\left(1-\sigma \left(\tilde{\Omega }\right)\right) \end{array}$$

Due to the effective integration of label information into the fusion embeddings by GAT, the generated pseudo-labels encompass information from both modalities while preserving the semantic relevance of the original modalities. This ensures that the subsequently generated hash codes are more discriminative.

### 3.3. Multiscale Label Area Hybrid Network (MLAH)

**Figure 3:**
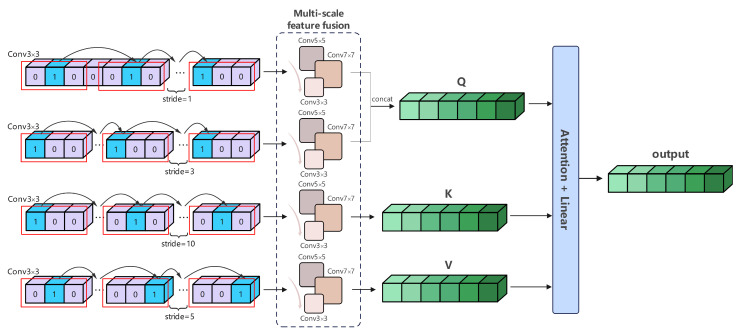
Multiscale Label Area Hybrid Network (MLAH) consists of a feature extraction module followed by four hierarchical multiscale attention modules, which are ultimately integrated through weighted fusion.

Label information, akin to text, encompasses a wealth of feature information. In cross-modal hash retrieval tasks, labels can serve as a complement to text, compensating for the lack of rich semantic features in text. Unlike the aforementioned method, GAT primarily utilizes labels as a form of supervisory information to guide the transformation of fused features into pseudo-labels, whereas the label network considers labels as a new modality of information. In cross-modal hash retrieval tasks, a single image sample corresponds to multiple different labels, naturally leading us to consider the multiscale information within labels. Moreover, due to the sparsity and diversity of label information distribution, the truly useful information is often nonadjacent. To address these issues, we have designed a multiscale area hybrid module, as shown in Figure 3, to establish connections between nonadjacent areas of label features while incorporating a self-attention mechanism to deepen internal semantic relevance. The overall algorithm is as follows:

(13) $$\begin{array}{c} \begin{array}{c} F_{z}^{k}=AutoEncoder\left(SoftMax\left(\frac{QK^{T}}{\sqrt{d_{k}}}\right)V\right)\;\\ s.t.\;Q=\mathrm{Concat}\left({\mathrm{AEMM}}_{1}\left(Z_{i}\right),{\mathrm{AEMM}}_{2}\left(Z_{i}\right)\right)\;\\ K=AEMM_{3}\left(Z_{i}\right)\;\;\\ V=AEMM_{4}\left(Z_{i}\right) \end{array} \end{array}$$

where $H_{z}^{k}$ represents the final label features obtained, $d_{k}$ denotes the modulation factor, AutoEncoder refers the automatic codec, and LKA stands for Local Kernel Alignment, as illustrated in Figure 2. Specifically, to reduce the presence of irrelevant information within tokens and make useful information more compact, we employ the Attention-Enhanced Multiscale Module (AEMM) to aggregate sparse information from the labels and perform multiscale regional blending. The specific algorithm for AEMM is as follows:

(14) $$\begin{array}{c} \begin{array}{c} AEMM_{j}=GMP\left(ReLU\left(LKA\left(ReLU\left(Conv1D\left(Z_{i}\right)\right)\right)\right)\right)\\ s.t.\;j\in \{1,2,3,4\}\;\;\;\;\;\;\;\;\;\;\;\;\;\;\;\;\;\;\;\;\;\;\;\;\;\;\;\;\;\;\;\; \end{array} \end{array}$$

where *j* indicates the use of different strides in one-dimensional convolution, $Z_{i}$ represents the use of different label samples, and GMP stands for Global Max Pooling.

We assign two smaller strides to *Q* through $AEMM_{1}$ and $AEMM_{2}$, and two larger strides to *K* and *V* through $AEMM_{3}$ and $AEMM_{4}$, respectively. This results in the acquisition of a query matrix containing compact information, as well as key and value matrices with significant information. The global dependencies of label information are obtained through the self-attention mechanism. Finally, we use H(·) to map the obtained multiscale label fusion features to the required hash code length:

(15) $$\begin{array}{c} H_{z}^{M}=H\left(F_{z}^{k}\right) \end{array}$$

### 3.4. Deep Text Feature Extraction Network (DTFEN)

**Figure 4:**
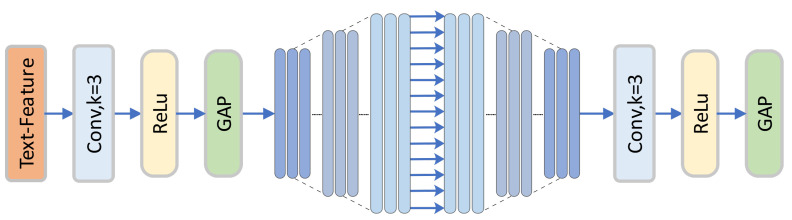
Deep Text Feature Extraction Network (DTFEN) comprises two deep extraction modules and an autoencoder.

Most cross-modal hash retrieval methods convert text into Bag-of-Words (BoW) vectors and then use a multilayer perceptron (MLP) for feature extraction. This approach leads to sparse information characteristics in feature embeddings, which are not conducive to generating compact text hash codes. Therefore, this paper adopts a fine-grained text feature extraction method to replace the traditional MLP or Transformer used in previous methods. Compared to the former, this approach can better learn text features by aggregating more sparse text features. Relative to the latter, our method reduces computational resources, accelerates computation speed, and does not significantly increase the number of parameters compared to the previous MLP. The network structure is shown in Figure 4, and the proposed text network is described as follows:

(16) $$\begin{array}{c} F_{y}^{k}=Stage_{2}\left(AutoEncoder\left(Stage_{1}\left(Y_{i}\right)\right)\right) \end{array}$$

(17) $$\begin{array}{c} \begin{array}{c} Stage_{i}=GAP(ReLU\left(Conv1D_{j}\left(\right)\right)\\ s.t.\;\;\;i,j\in \{1,2\} \end{array} \end{array}$$

where Conv$1D_{j}$ represents a one-dimensional convolution with a kernel size of 3×3, stride of 1, and padding of 1. $Stage_{i}$ indicates different feature extraction stages, and GAP stands for Global Average Pooling. To better blend fine-grained text representations, we utilize a deep module fusion with an autoencoder to obtain the text’s deep mixed features $F_{y}^{k}$. Finally, we use H(·) to transform text features into the required hash code length:

(18) $$\begin{array}{c} H_{y}^{M}=H\left(F_{y}^{k}\right) \end{array}$$

### 3.5. Hash Learning

The algorithm of TEGAH model is summarized in Algorithm 1. The following four loss functions are used to optimize the backpropagation process of TEGAH model. The four loss functions are described in detail below.
**Algorithm 1** Hash Learning algorithm of TEGAH**Input:**
Training set ${\left\{X_{i},Y_{i},Z_{i}\right\}}_{i=1}^{N}$, Binary code length *M*, Hyper-parameters *∂*, Query sets $Query_{i}$, Parameters for TEGAH.
**Output:**
Binary code $B_{i=x,y,z}^{M}$, Parameters $\Theta_{X}$, $\Theta_{Y}$ and $\Theta_{Z}$.
**Initialization:**
Initialize the parameters $\Theta_{X}$, $\Theta_{Y}$ and $\Theta_{Z}$, maximum iteration number epoch, mini-batch size 80.
1:**while** iter<epoch **do**2:    Compute $F_{x}^{k}$, $F_{y}^{k}$, $F_{z}^{k}$ features using Equations (3), (15) and (18) for the training set.3:    Compute GAT fusion features using Equation (11).4:    Calculate losses $L_{\mathrm{tri}}$, $L_{\mathrm{class}}$, $L_{\mathrm{quan}}$, $L_{\mathrm{wass}}$ and $L_{\mathrm{kl}}$ using Equations (12), (19), (22), (26) and (27).5:    Calculate approximate binary hash codes using query sets data.6:    Input to the trained TEGAH model.7:    Calculate binary hash codes using the function.8:**end while**
**return** the TEGAH model after training.


#### 3.5.1. Cosine Weighted Triplet Loss

To maintain similarity among hash codes from different modalities, this paper introduces a cosine-weighted triplet loss mechanism. This approach maps features from various modalities to a binary Hamming space that reflects similar semantic meanings, thereby facilitating efficient similarity measurement and retrieval. The hash function is trained using triplet samples $\left\{{\tilde{b}}_{i}^{x},{\tilde{b}}_{k}^{y-},{\tilde{b}}_{j}^{y+}\right\}$, each consisting of an anchor and two positive samples derived from both identical and distinct modalities. A sample is defined as positive if it shares at least one label with the anchor; otherwise, it is considered negative. The aim of the cosine-weighted triplet loss during training is to decrease the cosine distance between hash codes of the same modality while increasing the distance between those of different modalities. By adjusting the weights of the weighted terms, the model learns a mapping function that preserves semantic similarity across modalities within the hash space. Additionally, the introduction of normalized weighting factors optimizes structural similarity within the multilabel semantic space. The aforementioned analysis yields the following definition:

(19) $$\begin{array}{c} L_{tri}=L_{tri}^{i->t}+L_{tri}^{t->i} \end{array}$$

where $L_{tri}^{i->t}$ represents the cosine-weighted triplet loss from images to text and $L_{tri}^{t->i}$ represents the cosine-weighted triplet loss from text to images, which is similar to $L_{tri}^{i->t}$ and will not be elaborated further here. Among them, $L_{tri}^{i->t}$ can be defined as follows:

(20) $$L_{tri}^{i->t}=\sum_{i,j,k}\tau_{jk}\max(({\lambda^{x}}_{i,k})-{\lambda^{x}}_{i,j}+m,0)$$

where *m* represents the margin coefficient, which adjusts the threshold of similarity for the triplet loss, η denotes the regularization coefficient, $\tau_{jk}$ represents the weight factor, $v_{j}$ and $v_{k}$ indicate the similarity between the labels of the positive and negative samples from different modalities with the anchor, computed through cosine similarity, and $\lambda_{i,k}^{x}$ and $\lambda_{i,j}^{x}$ represent the similarity matrices obtained through cosine similarity calculations. The definition is as follows:

(21) $$\begin{array}{c} \tau_{jk}=\frac{2^{\upsilon_{j}}-2^{\upsilon_{k}}}{\eta }\\ \lambda_{i,k}^{x}=\cos\left({\tilde{b}}_{i}^{x},{\tilde{b}}_{k}^{y-}\right)\\ \lambda_{i,j}^{x}=\cos({\tilde{b}}_{i}^{x},{\tilde{b}}_{j}^{y+}) \end{array}$$

where ${\tilde{b}}_{i}^{x}$ and ${\tilde{b}}_{i}^{y}$ represent the hash codes that have been processed by the tanh activation function but have not yet been binarized. These continuous-valued representations serve as intermediate outputs. In contrast, the hash codes without the tilde symbol (e.g., $b_{i}^{x}$) denote the final binarized codes, which are obtained after applying the sign function.

#### 3.5.2. Label Distillation Loss

To optimize the structural semantics of multilabel data, relying solely on cosine-weighted triplet loss is insufficient. It is crucial to maintain consistency in the semantic space between labels and hash codes. Therefore, we employ label distillation loss to preserve the semantic relevance between hash codes and labels. The definition of label distillation loss is as follows:

(22) $$\begin{array}{c} KL_{loss}=\frac{1}{2}\left(KL_{L,H}+KL_{H,L}\right)+KL_{S} \end{array}$$

where $KL_{H,L}$ denotes the distillation loss from hash codes to labels, $KL_{L,H}$ represents the distillation loss from labels to hash codes, $KL_{S}$ indicates the mean squared error of similarity. The definitions are as follows:

(23) $$\begin{array}{c} KL_{L,H}=\frac{1}{BN}\sum_{i=1}^{B}\sum_{j=1}^{N}\max\left(0,S_{L,H}\right) \end{array}$$

(24) $$\begin{array}{c} KL_{H,L}=\frac{1}{BN}\sum_{i=1}^{B}\sum_{j=1}^{N}\max\left(0,S_{H,L}\right) \end{array}$$

(25) $$\begin{array}{c} \begin{array}{c} KL_{S}=\frac{1}{BN}\sum_{i=1}^{B}\sum_{j=1}^{N}(sim(H_{i},L_{j})-sim\left(L_{i},H_{j}\right){)}^{2}\\ s.t.\;S_{L,H}=sim\left(L_{i},H_{j}\right)-sim\left(H_{i},L_{j}\right)\\ \;\;\;\;\;S_{H,L}=sim\left(H_{i},L_{j}\right)-sim\left(L_{i},H_{j}\right) \end{array} \end{array}$$

where $S_{L,H}$ and $S_{H,L}$, respectively, represent the similarity matrices from hash codes to labels and from labels to hash codes, *B* represents the batch size, and *N* denotes the number of samples used for training.

#### 3.5.3. Quantization Loss

Quantization loss, through learning a hash function, maps real-valued features to binary hash codes, aiming to preserve data similarity as much as possible. $b_{{*}_{i,j}}^{M}$ represents a hash code of length *M*, *N* denotes the number of samples to be learned in each batch, and *x*, *y*, and *z* represent images, text, and labels, respectively. We define $b_{{*}_{i,j}}^{M},i\in B,j\in N,$$*\in \{H_{X}^{M},H_{Y}^{M},H_{Z}^{M}\}$. We employ the squared L2 norm loss to measure the distance between discrete hash codes and continuous values, training the model by minimizing the distance or discrepancy between real-valued features and their corresponding binary hash codes. By calculating the Hamming distance between binary hash codes, semantically similar cross-modal data can be found and their similar structure can be maintained. The following definition can be obtained:

(26) $$\begin{array}{c} \begin{array}{c} L_{quan}=\frac{1}{BN}\sum_{i=1}^{B}\sum_{j=1}^{N}\left(b_{x_{i,j}}^{M}+b_{y_{i,j}}^{M}+b_{z_{i,j}}^{M}\right)\;\\ b_{x}^{M}={∥\mathrm{sign}\left(H_{x}^{M}\right)-H_{x}^{M}∥}_{2}^{2}\;\\ b_{y}^{M}={∥\mathrm{sign}\left(H_{y}^{M}\right)-H_{y}^{M}∥}_{2}^{2}\;\\ b_{z}^{M}={∥\mathrm{sign}\left(H_{z}^{M}\right)-H_{z}^{M}∥}_{2}^{2} \end{array} \end{array}$$

#### 3.5.4. Wasserstein Loss

Wasserstein Loss: The Wasserstein distance [45], in mathematics, refers to a distance function between probability distributions on a given metric space *M*. By incorporating it into the TEGAH framework, it is utilized to balance differences between various modalities, aiming to achieve effective optimization for cross-modal hash retrieval. The definition of Wasserstein loss is as follows:

(27) $$\begin{array}{c} \begin{array}{c} L_{wass}=EMD\left(P_{i},P_{j}\right)\\ \;\;\;\;\;\;\;\;=\underset{\gamma (x,y)\in \Pi }{\inf}\sum_{x,y}∥H_{x}^{M}-H_{y}^{M}∥\gamma \left(H_{x}^{M},H_{y}^{M}\right)\\ \;\;\;\;\;\;\;\;=\underset{\gamma (x,y)\in \Pi }{\inf}E_{(x,y)∼\gamma }∥H_{x}^{M}-H_{y}^{M}∥ \end{array} \end{array}$$

where $P_{i}$ and $P_{j}$ are two probability distributions, *X* and *Y* are random variables in $P_{i}$ and $P_{j}$, $\left|\right|H_{x}^{M}-H_{y}^{M}\left|\right|$ denotes the distance between the image modality and the text modality hash code, which is measured here using the Euclidean distance, and γ(·) denotes the minimum of all distances. We introduce the Wasserstein distance into cross-modal hash retrieval to better compensate for the differences between modalities. Finally, our proposed TEGAH method uses cosine-weighted ternary loss, label distillation loss, quantization loss, Wasserstein distance loss, and the total Loss can be computed by the following equation:

(28) $$\begin{array}{c} L_{total}=\alpha (L_{tri}^{i->t}+L_{tri}^{t->i})+L_{class}+L_{quan}+L_{wass}+L_{kl} \end{array}$$

where α is the hyperparameter to balance the cosine-weighted triad loss with other losses, and in our experiments α is taken to be 10.

### 3.6. Baseline Setting

In our experiments, we selected 14 state-of-the-art cross-modal hash retrieval methods for comparison, including DCMH [5], CMHH [6], AGAH [7], CPAH [8], DADH [14], SCAHN [17], DCHUC [21], MESDCH [25], SCCGDH [20], MIAN [19], GCDH [42], DAPH [18], MAFH [24], and DSPH [23]. For all methods, we utilized the same experimental setup and maintained consistency in the division of datasets, retrieval sets, and query sets across all approaches, aligning them with our experimental configurations.

## 4. Experiments

To validate the effectiveness of our proposed Text-Enhanced Graph Attention Transformer for Hash-based Cross-Modal Retrieval (TEGAH) method, we carried out comprehensive experiments on three public multimodal retrieval datasets: MIRFLICKR-25K, NUS-WIDE, and MS-COCO. In the following sections, we elaborate on the experimental results of several state-of-the-art algorithms compared to our approach. Furthermore, we provide detailed descriptions of the three datasets used for experimental training, explain the experimental details of TEGAH, evaluate TEGAH’s performance metrics, and describe the experimental setup.

### 4.1. Datasets

In the experiments of this paper, we employ the same sampling strategy across three large-scale multilabel datasets: MIRFLICKR-25K (https://press.liacs.nl/mirflickr (accessed on 17 October 2024)) [46], NUS-WIDE (https://www.kaggle.com/datasets/xinleili/nuswide (accessed on 17 October 2024)) [47], and MS-COCO (https://cocodataset.org/ (accessed on 17 October 2024)) [48]. Each dataset is divided into training sets, test sets, and retrieval sets. For different datasets, images and texts are processed in the same manner, with the input network’s image resolution set to 224 × 224. Text is represented using Bag-of-Words (BoW) encoding. Specific details about the division of datasets and the dimensions of text feature encodings are presented in Table 1.

### 4.2. Evaluation Criteria

In our work, we employ Mean Average Precision (mAP) and the Precision-Recall curve (PR curve) as evaluation metrics for our experiments. These metrics are detailed as follows.

#### 4.2.1. Mean Average Precision (mAP)

mAP is a method used to assess the performance of retrieval systems, measuring the average level of accuracy within the retrieval results. The mAP value represents the average precision, assessing whether the modality retrieved matches the query modality category, commonly used to evaluate the performance of cross-modal retrieval algorithms. Given a set of query data *Q* and *N* retrieval results, the mean average precision can be expressed as:

(29) $$\begin{array}{c} mAP=\frac{1}{QR}\sum_{q=1}^{Q}\sum_{i=1}^{N}P(i)\delta (i) \end{array}$$

where P(i) denotes the precision of the top *i* retrieval results, and δ(i)=1 equals 1 if the retrieval result is relevant to the query, and 0 otherwise, i.e., δ(i)=0,Q represents the number of queries initiated, and R represents the size of the entire search set.

The mAP serves as a metric to assess the performance of retrieval systems, aiding in the evaluation of the accuracy of retrieval outcomes and the effectiveness of the retrieval system. In our work, we utilize the mAP@all evaluation metric, where “all” refers to the size of the entire retrieval set.

#### 4.2.2. Precision-Recall (PR) Curve

The PR curve represents the precision of the retrieved ranked list at different recall levels.

### 4.3. Experimental Details

In this paper, we employ a model pre-trained on ImageNet-1K as the backbone network for image processing, extract textual features using a deep text network, and utilize a GAT to optimize cross-modal feature fusion. Additionally, a label network supplements textual semantic features. The input to our framework’s GAT consists of two adjacency matrices constructed from label information optimized through cosine similarity. Our TEGAH framework is implemented in PyTorch version 2.1.0, with Python version 3.10 and CUDA version 12.1. All experiments were conducted on a computer equipped with an NVIDIA RTX-3090 Ti GPU and 128 GB RAM. In our experiments, the learning rate for the image network was set between 10 × 10^−5^ and 10 × 10^−6^, while for the GAT network, text network, and label network, it ranged from 10 × 10^−4^ to 10 × 10^−5^. The batch size was set at 80, and the number of epochs at 300. We optimized the image and GAT networks using AdamW optimizer and the text and label networks using the Adam optimizer.

### 4.4. Analysis of Experimental Results

#### 4.4.1. Comparison with the Baselines

**Figure 5:**
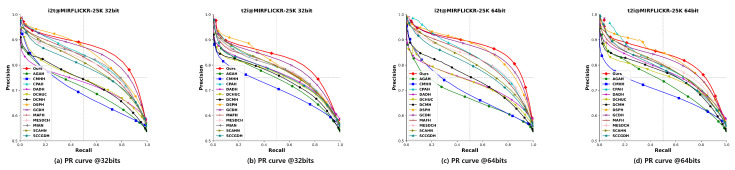
Results of PR curves of 32 bits and 64 bits on MIRFLICKR-25K dataset.

**Figure 6:**
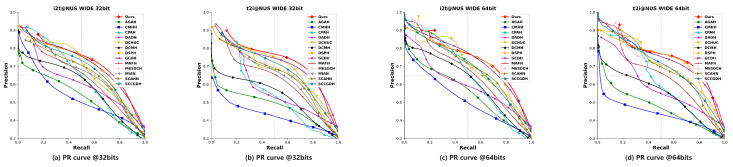
Results of PR curves of 32 bits and 64 bits on NUS-WIDE dataset.

**Figure 7:**
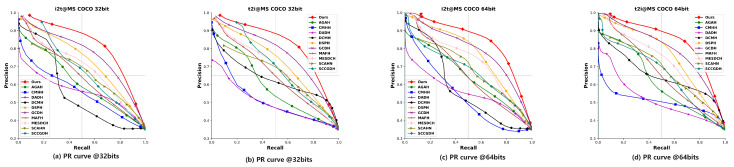
Results of PR curves of 32 bits and 64 bits on MS-COCO dataset.

To assess the effectiveness and advancement of our proposed TEGAH method, we conducted a comparative analysis with 14 state-of-the-art (SOTA) cross-modal hash retrieval methods in terms of mAP values and PR curves. This comparison encompasses two evaluation tasks: using images to retrieve text, denoted as “Image-to-Text” (I2T), and using text to retrieve images, denoted as “Text-to-Image” (T2I). Table 2 present the mAP comparison results for each method across three different datasets with 16, 32, and 64-bit hash codes. Compared to the second-best method GCDH, our TEGAH method shows a maximum performance increase of 1.7% and an average increase of 1.35% on the MIRFLICKR-25K dataset, a maximum increase of 2.3% and an average increase of 0.75% on the NUS-WIDE dataset, and a notable maximum increase of 4.4% and an average increase of 3.8% on the MS-COCO dataset. The significant improvement on the MS-COCO dataset may be attributed to its larger number of labels compared to the other two datasets (MIRFLICKR-25K has 24 category labels, while NUS-WIDE has only 10), as a limited number of category labels can affect the multiscale feature information extracted by the Multiscale Label Area Hybrid Network (MLAH) and lead to sparser features when fusing modal features for the Graph Attention Module, ultimately impacting retrieval performance. Although TEGAH’s performance on the other two datasets (MIRFLICKR-25K and NUS-WIDE) did not reach the level achieved on the MS-COCO dataset, the results indicate that our TEGAH method can still effectively learn multiscale label features and optimize text feature extraction in scenarios of sparse text feature information and limited label category information. By employing the Multiscale Label Area Hybrid Network and the Deep Text Feature Extraction Network, TEGAH can compensate for the scarcity of textual information and category labels. Furthermore, the Graph Attention Feature Fusion Module enables the alignment and fusion of different modal information, utilizing learned implicit information to bridge the information gap between modalities, thereby optimizing the generation of final hash codes and enhancing retrieval performance. Figure 5, Figure 6 and Figure 7 showcase the Precision-Recall (PR) curves for hash code lengths of 32 and 64 bits. It is observable that, in most instances, the PR curve trends of our proposed TEGAH method outperform those of other methods across the three datasets. On the NUS-WIDE dataset, our method surpasses the second-best method GCDH in T2I performance, but slightly lags behind GCDH in I2T performance. This discrepancy can be attributed to the lesser number of category labels used in the NUS-WIDE dataset, which results in an insufficient number of features for the adjacency matrix required by the MLAH and the GAFM. Consequently, the GAFM cannot fully utilize the feature information from different modalities for alignment and fusion, thereby affecting the generation of hash codes. This highlights the importance of adequate category labels and adjacency matrix features in enhancing the effectiveness of cross-modal feature fusion and alignment, which are critical for generating distinctive and accurate hash codes.

#### 4.4.2. Ablation Experiments

For cross-modal retrieval tasks, our proposed method performs two evaluation tasks: “I2T” for retrieving text using images and “T2I” for retrieving images using text. In order to validate the effectiveness of our proposed TEGAH method, we conducted extensive experiments on three public datasets with the following details:

Table 3 outlines the design of our ablation experiments, featuring eight variants: (a) ‘baseline’ refers to the base model, where the GAFM, MLAH, and DTFEN modules are removed from the final network, while all other parameter settings are retained. (b) ‘TEGAH’ represents the complete model, incorporating the GAFM, MLAH, and DTFEN modules. (c) ‘TEGAH-V1’ adds only the MLAH module to the baseline model. (d) ‘TEGAH-V2’ introduces only the GAFM module into the baseline model. (e) ‘TEGAH-V3’ includes only the DTFEN module in the baseline model. (f) ‘TEGAH-V4’ integrates both the GAFM and MLAH modules into the baseline model. (g) ‘TEGAH-V5’ integrates both the DTFEN and MLAH modules into the baseline model. (h) ‘TEGAH-V6’ incorporates both the DTFEN and GAFM modules into the baseline model. Table 4 presents the results of ablation studies. The outcomes from experiments TEGAH-V3, TEGAH-V5, and TEGAH-V6 indicate that the incorporation of the DTFEN notably enhances the text feature extraction, particularly yielding better results for hash codes of lower bit lengths. This improvement in text feature extraction concurrently elevates the performance of the image feature extraction network to a certain extent. Furthermore, the results from TEGAH-V1, TEGAH-V4, and TEGAH-V5 demonstrate significant improvements in “Text-to-Image” (T2I) retrieval following the integration of the MLAH. This suggests that treating label information as a modality for multiscale weighted fusion can effectively compensate for the scarcity of textual feature information. Additionally, the introduction of the GAFM, as evidenced by the results from TEGAH-V2, TEGAH-V4, and TEGAH-V6, leads to enhanced retrieval performance compared to the baseline. This enhancement indicates that GAFM can effectively integrate and align features from different modalities, reinforcing their representation and mitigating information loss in hash codes. Finally, the comprehensive performance improvement observed in the results from TEGAH-V0, where all three modules were utilized, validates the efficacy and rationale of our TEGAH framework.

#### 4.4.3. Top-5 Retrieval Outcomes

**Figure 8:**
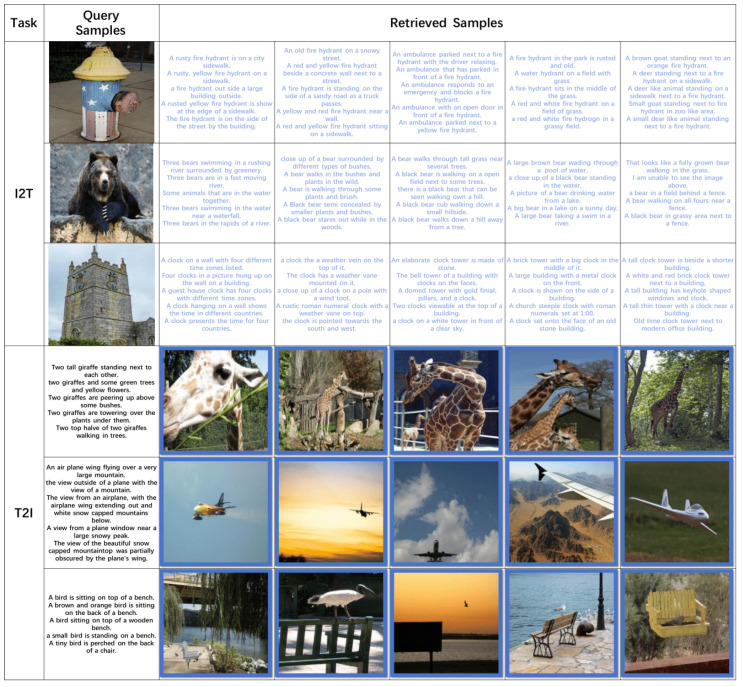
Utilizing our TEGAH framework, original samples are encoded and subjected to retrieval within the MS-COCO dataset, employing 64-bit hash codes to ascertain the top 5 results. Samples returned and denoted with a blue marker signify relevance to the query sample.

To showcase the effective retrieval capability of our introduced TEGAH approach, we employed the MS-COCO dataset for Hamming ranking, as illustrated in Figure 8. The retrieval instances obtained through our TEGAH approach are all pertinent. This suggests that the TEGAH approach can markedly improve the performance of the text feature extraction network. Moreover, the Multiscale Label Area Hybrid Network can, to some extent, compensate for the scarcity of textual information. Consequently, through the Graph Attention Feature Fusion Module, it is possible to better integrate the semantic information of multilabels, generating more distinctive hash codes. This ability to accurately retrieve relevant results underscores TEGAH’s effectiveness in addressing the challenges of cross-modal hash retrieval, particularly in bridging the semantic gap between different modalities and improving the richness of textual features for more accurate and efficient search outcomes.

#### 4.4.4. Visualization Results

**Figure 9:**
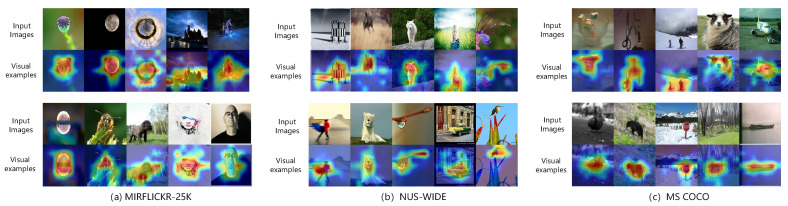
The results of visualization of 10 images randomly selected in three datasets using the Grad-CAM method.

To further validate the capability of the image feature extraction network in capturing global information, Figure 9 presents several examples of feature visualization using our proposed TEGAH method. Across three datasets, we selected 10 images each and visualized their feature maps using the Grad-CAM method, specifically visualizing the outputs before the LayerNorm layer of the last encoder block of the image feature extractor. GradCAM visualizations highlight the regions of the image feature extraction network that may influence the classification decision, which often contain key descriptive elements. For instance, on the MS-COCO and NUS-WIDE datasets, certain images encompass two identical objectives, and the image feature extraction network within the TEGAH method can precisely capture both objectives. This demonstrates TEGAH’s effectiveness not only in feature extraction but also in ensuring that the extracted features are meaningful and relevant to the image content, thereby enhancing the accuracy of subsequent retrieval tasks.

## 5. Discussion

Although TEGAH has demonstrated strong performance, there remains room for improvement in the image modality. At present, the potential of image feature extraction and modality alignment has not been fully realized in certain complex scenarios, which may negatively impact the overall retrieval performance.

Furthermore, with the advancement of large language models (LLMs), these models have evolved into systems capable of processing multimodal information. This development presents new opportunities for enhancing both image and text modalities. Moving forward, we will shift our focus towards improving existing cross-modal retrieval methods. We will explore novel approaches for optimizing the extraction and integration of image features without relying on labeled information. Additionally, we will leverage the capabilities of LLMs to enrich text modality features, with the goal of generating more distinctive and robust hash codes.

While the current approach relies on labeled data, our future work will aim to reduce this dependency. We plan to investigate weakly-supervised, semi-supervised, and unsupervised learning methods to mitigate the reliance on high-quality labeled data, thereby making the retrieval methods more adaptable and applicable to a broader range of real-world scenarios. Moreover, we will validate the performance of these improved methods on large-scale unsupervised datasets to ensure their generalizability and scalability across different data environments.

## 6. Conclusions

In this paper, we propose a new Text-Enhanced Graph Attention Hashing for the Cross-Modal Retrieval (TEGAH) framework. First, we use the deep text feature extraction network to extract deep features of text information so that we can directly improve the extracted text features without changing the text features and improve the retrieval effect. Secondly, we regard label information as a mode and propose a multiscale label region hybrid network, which can supplement the modal features of text and alleviate the information gap when text information is scarce. Finally, in order to integrate the features of different modes, TEGAH uses GAT to learn a set of interdependent modal features, and optimizes the learned features for modal alignment and fusion, preserving the common features of different modes, bridging the information gap between different modes, and generating more distinctive hash codes. A large number of experiments on MIRFLICKR-25K, NUS-WIDE, and MS-COCO datasets prove that TEGAH method has good retrieval performance. A large number of experiments on the MIRFLICKR-25K, NUS-WIDE, and MS COCO datasets demonstrate that the TEGAH method achieves outstanding retrieval performance and significantly outperforms existing cross-modal hashing methods.

## Figures and Tables

**Table 1:** Characterization statistics for the three benchmark datasets.

Dataset Details	MIRFLICKR-25K	NUS-WIDE	MS-COCO
Dataset Size	20,015	186,577	122,218
Training Size	10,000	10,500	10,000
Retrieval Size	18,015	184,477	117,218
Query Size	2000	2100	5000
Number of Categories	24	10	80
Dim of Text Features	1386	1000	2026

**Table 2:** The MAP protocols on MIRFLICKR25K, NUS-WIDE, and MS-COCO (MAP@ALL). Results are indicated in bold. ‘/’ denotes unavailable results, and ‘*’ indicates results cited from the original paper.

Task	Method	MIRFLICKR-25K			NUS-WIDE			MS-COCO		
		16 bits	32 bits	64 bits	16 bits	32 bits	64 bits	16 bits	32 bits	64 bits
I2T	DCMH [5]	0.7323	0.7432	0.7502	0.5248	0.6000	0.6197	0.5179	0.5314	0.5472
	CMHH [6]	0.6863	0.6901	0.6887	0.5233	0.5171	0.5236	0.5530	0.5461	0.4714
	AGAH [7]	0.7006	0.7241	0.6912	0.3945	0.4107	0.4258	0.5501	0.5515	0.5518
	CPAH [8]	0.8063	0.8237	0.8305	0.5686	0.6207	0.6342	0.5949	0.6426	0.6448
	DADH [14]	0.7333	0.7449	0.7496	0.5953	0.6084	0.6030	0.5750	0.5788	0.5755
	SCAHN [17]	0.7828	0.7942	0.8021	0.6550	0.6580	0.6744	0.6479	0.6426	0.6431
	DCHUC [21]	0.7358	0.7464	0.7427	0.6159	0.6460	0.6755	0.5282	0.5489	0.5338
	MESDCH [25]	0.7898	0.8032	0.8153	0.6607	0.6832	0.6968	0.6590	0.6960	0.7212
	SCCGDH [20]	0.7748	0.7949	0.7933	0.6770	0.6931	0.6977	0.6044	0.6351	0.6647
	MIAN [19]	0.8044	0.8178	0.8183	0.6303	0.6433	0.6374	0.5856	0.6121	0.6131
	GCDH [42]	0.8373	0.8545	0.8630	**0.7136**	**0.7263**	**0.7424**	0.7268	0.7630	0.7826
	DAPH * [18]	/	/	/	/	0.6840	0.6930	0.6870	0.7180	/
	MAFH [24]	0.7981	0.8168	0.8263	0.6367	0.6422	0.6582	0.6044	0.6689	0.6871
	DSPH [23]	0.8016	0.8301	0.8446	0.6847	0.7015	0.7125	0.6864	0.7493	0.7704
	Ours	**0.8484**	**0.8665**	**0.8740**	0.7052	0.7236	0.7356	**0.7542**	**0.8021**	**0.8219**
T2I	DCMH [5]	0.7554	0.7716	0.7788	0.5545	0.5903	0.5957	0.5508	0.5883	0.6049
	CMHH [6]	0.6809	0.7134	0.7012	0.4795	0.4541	0.4668	0.4847	0.4980	0.5053
	AGAH [7]	0.6873	0.7496	0.7478	0.4344	0.3980	0.4382	0.5012	0.5146	0.5191
	CPAH [8]	0.7947	0.8064	0.8082	0.5605	0.5686	0.6053	0.5891	0.6384	0.6413
	DADH [14]	0.7641	0.7748	0.7813	0.5631	0.5609	0.5711	0.4767	0.4819	0.4921
	SCAHN [17]	0.7845	0.7956	0.7997	0.6692	0.6715	0.6795	0.6470	0.6430	0.6396
	DCHUC [21]	0.7522	0.7712	0.7708	0.6356	0.6795	0.7019	0.5220	0.5269	0.5185
	MESDCH [25]	0.7741	0.7898	0.7991	0.6662	0.6840	0.6977	0.6345	0.6737	0.7019
	SCCGDH [20]	0.7622	0.7785	0.7903	0.6759	0.7072	0.7115	0.5949	0.6427	0.6475
	MIAN [19]	0.7947	0.8013	0.8082	0.6486	0.6685	0.6586	0.5459	0.5997	0.5940
	GCDH [42]	0.8103	0.8230	0.8319	0.7195	0.7348	0.7474	0.7219	0.7597	0.7845
	DAPH * [18]	/	/	/	/	0.6770	0.6890	0.7030	0.7300	/
	MAFH [24]	0.7841	0.7982	0.8006	0.6357	0.6480	0.6542	0.5963	0.6733	0.6912
	DSPH [23]	0.7972	0.8133	0.8351	0.7025	0.7177	0.7315	0.6921	0.7520	0.7714
	Ours	**0.8238**	**0.8406**	**0.8481**	**0.7403**	**0.7578**	**0.7665**	**0.7593**	**0.8044**	**0.8263**

**Table 3:** Ablation experiment settings for each module. ✔ indicates that the module is used, ✗ indicates that the module is not used.

	MLAH	GAFM	DTFEN
TEGAH	✔	✔	✔
TEGAH-V1	✔	✗	✗
TEGAH-V2	✗	✔	✗
TEGAH-V3	✗	✗	✔
TEGAH-V4	✔	✔	✗
TEGAH-V5	✔	✗	✔
TEGAH-V6	✗	✔	✔

**Table 4:** Ablation experiment results.

Task	Method	MIRFLICKR-25K			NUS-WIDE			MS-COCO		
		16 bits	32 bits	64 bits	16 bits	32 bits	64 bits	16 bits	32 bits	64 bits
	baseline	0.8219	0.8500	0.8620	0.6868	0.7174	0.7312	0.6816	0.7526	0.7859
	TEGAH-V1	0.8225	0.8506	0.8620	0.6822	0.7176	0.7436	0.6987	0.7502	0.7882
	TEGAH-V2	0.8261	0.8537	0.8696	0.6980	0.7187	**0.7442**	0.6845	0.7523	0.7913
	TEGAH-V3	0.8441	0.8571	0.8644	**0.7053**	0.7155	0.7283	0.7287	0.7822	0.8054
	TEGAH-V4	0.8260	0.8513	0.8632	0.6960	0.7219	0.7415	0.6874	0.7536	0.7900
	TEGAH-V5	0.8480	0.8592	0.8643	0.7044	0.7119	0.7268	0.7322	0.7841	0.8063
	TEGAH-V6	0.8476	0.8575	0.8651	0.7036	0.7107	0.7290	0.7346	0.7817	0.8086
I2T	TEGAH	**0.8484**	**0.8665**	**0.8740**	0.7052	**0.7236**	0.7356	**0.7542**	**0.8021**	**0.8219**
	baseline	0.7794	0.8095	0.8292	0.6844	0.7189	0.7303	0.6804	0.7448	0.7801
	TEGAH-V1	0.7981	0.8156	0.8339	0.6911	0.7237	0.7430	0.6970	0.7471	0.7842
	TEGAH-V2	0.7836	0.8089	0.8262	0.7092	0.7202	0.7418	0.6882	0.7439	0.7860
	TEGAH-V3	0.8108	0.8267	0.8368	0.7254	0.7401	0.7498	0.7227	0.7812	0.8069
	TEGAH-V4	0.7956	0.8110	0.8318	0.7004	0.7288	0.7464	0.6822	0.7475	0.7850
	TEGAH-V5	0.8176	0.8313	0.8410	0.7234	0.7367	0.7519	0.7268	0.7828	0.8094
	TEGAH-V6	0.8158	0.8295	0.8397	0.7278	0.7377	0.7466	0.7312	0.7812	0.8126
T2I	TEGAH	**0.8238**	**0.8406**	**0.8481**	**0.7403**	**0.7578**	**0.7665**	**0.7593**	**0.8044**	**0.8263**

## Data Availability

The MIRFLICKR-25K Dataset in this study is openly and freely available at https://press.liacs.nl/mirflickr (accessed on 21 October 2024). The NUS-WIDE dataset in this study are openly and freely available at https://www.kaggle.com/datasets/xinleili/nuswide (accessed on 21 October 2024). The MS-COCO 2014 dataset in this study are openly and freely available at https://cocodataset.org (accessed on 21 October 2024). Our code is available at https://github.com/ShiShuMo/TEGAH (accessed on 21 October 2024).
